# DSCAM‐AS1 regulates the G_1_/S cell cycle transition and is an independent prognostic factor of poor survival in luminal breast cancer patients treated with endocrine therapy

**DOI:** 10.1002/cam4.1603

**Published:** 2018-11-14

**Authors:** Wei Sun, An‐Qi Li, Ping Zhou, Yi‐Zhou Jiang, Xi Jin, Yi‐Rong Liu, Ya‐Jie Guo, Wen‐Tao Yang, Zhi‐Ming Shao, Xiao‐En Xu

**Affiliations:** ^1^ Department of Breast Surgery Fudan University Shanghai Cancer Center Shanghai China; ^2^ Cancer Institute Fudan University Shanghai Cancer Center Shanghai China; ^3^ Department of Oncology Shanghai Medical College Fudan University Shanghai China; ^4^ Department of Pathology Fudan University Shanghai Cancer Center Shanghai China; ^5^ Department of Breast Surgery The Third Hospital of Nanchang Nanchang China; ^6^ Institutes of Biomedical Sciences Fudan University Shanghai China

**Keywords:** DSCAM‐AS1, endocrine therapy, long noncoding RNA, luminal breast cancer, prognostic factor

## Abstract

DSCAM‐AS1 is one of the few intensively studied lncRNAs with high specific expression in luminal breast cancer. It is directly regulated by estrogen receptor α (ERα) and plays vital roles in tumor proliferation, invasion, and tamoxifen resistance. However, the detailed function of DSCAM‐AS1 in tumor progression and its clinical significance remain unclear. We reveal that DSCAM‐AS1 regulates cell proliferation and colony formation by inducing the G1/S transition. RNA‐seq analysis demonstrated that DSCAM‐AS1 participates in crucial biological processes, including DNA replication, the G1/S phase transition, sister chromatid cohesion, chromosome segregation, protein localization to the chromosome and DNA recombination. Most importantly, in the retrospectively registered clinical analysis, high expression of DSCAM‐AS1 is a poor prognostic factor in patients with luminal breast cancer treated with endocrine therapy. In conclusion, DSCAM‐AS1 is a promising clinical therapeutic target that may prolong survival of luminal breast cancer patients treated with endocrine therapy.

## 1 BACKGROUND

Breast cancer is one of the most common cancers in the world with high heterogeneity.^1^ Using microarray and hierarchical clustering analysis, breast cancer can be divided into 5 “intrinsic” subtypes: luminal A, luminal B, Her‐2 overexpression, basal‐like and normal‐like, each subtype has its unique clinicopathologic features and treatment strategy.^2^

In recent years, an increasing number of long noncoding RNAs (lncRNAs) has been revealed to play fundamental roles in tumorigenesis and the progression of breast cancer. For example, lncRNA‐HOTAIR is essential in breast cancer metastasis. HOTAIR is highly expressed in metastatic tumors and induces Polycomb repressive complex 2 (PRC2)‐mediated epigenetic changes to promote tumor metastasis.^3^ NKILA, which is regulated by NF‐κB, binds to NF‐κB/IκB to inhibit its activation. Weak expression of NKILA is correlated with breast cancer metastasis and indicates a poor prognosis.^4^ BCAR4 is highly expressed in triple negative (especially metastatic) breast cancer. It is regulated by the noncanonical Hedgehog signaling pathway and binds to SNIP1 and PNUTS to regulate the activation of GLI2 target genes. Down‐regulation of BCAR4 inhibits breast cancer metastasis.^5^ LINK‐A mediates HIF1α phosphorylation by BRK and LRRK2 to stabilize HIF1α. LINK‐A expression and activation of its signaling pathway correlate with triple negative breast cancer (TNBC), promoting breast cancer glycolysis reprogramming and tumorigenesis.^6^ Owing to its high tissue specificity, lncRNAs may be distributed differently with disparate roles in various tissues. Tumor progression of different breast cancer subtypes is likely mediated by different lncRNAs. Although an appreciable number of breast cancer‐related lncRNAs has been investigated, most are associated with TNBC. lncRNA studies on luminal breast cancer, which account for two‐thirds of all breast tumors, are rare.^7^

DSCAM‐AS1 was originally described by Liu. It is transcribed from a gene located on chromosome 21q22.3 (GRCh38/hg38) with a full length of approximately 1.4 kb.^8^ In 2015, Miano et al^9^ analyzed the RNA‐seq data from MCF‐7 cells in the absence of estrogen. A list of 133 Apo‐ER‐regulated lncRNAs (AER‐lncRNAs) was identified, among which, the most abundant lncRNA, DSCAM‐AS1, was further investigated. They found that DSCAM‐AS1 was a major discriminant of the luminal subtype (ER+) of breast cancer and is not expressed in basal‐like breast cancer. DSCAM‐AS1 is directly regulated by ER and down‐regulation of DSCAM‐AS1 induced growth arrest and of the expression of EMT markers. In 2016, Yashar et al found the same lncRNA after analyzing the TCGA RNA‐seq cohort, which included 947 breast cancer samples.^10^ They demonstrated that DSCAM‐AS1 interacted with hnRNPL to mediate tumor progression and tamoxifen resistance. Hence, benefiting from the highest expression and crucial role in tumor progression and endocrine therapy resistance, DSCAM‐AS1 became one of the earliest intensively studied luminal breast cancer‐related lncRNAs.

However, before DSCAM‐AS1 can be considered a promising therapy target, several questions need to be addressed. For example, What is the detailed function of DSCAM‐AS1 in tumor progression? What biological processes and signaling pathways does DSCAM‐AS1 participate in? And, most importantly, what is the clinical significance of DSCAM‐AS1 in luminal breast cancer? In the current study, we aimed to reveal the role of DSCAM‐AS1 in luminal breast cancer progression and prognosis by utilizing cellular experiments and analyzing a large cohort of luminal breast cancer samples.

## 2 METHODS

### 2.1 Cell lines and cell culture

MCF‐7 (ATCC^®^ HTB‐22^™^) and T‐47D (ATCC^®^ HTB‐133^™^) cell lines were obtained from the ATCC. Both cell lines were cultured in RPMI1640 medium supplemented with 10% fetal calf serum (FCS), penicillin (100 units/mL), and streptomycin (100 μg/mL) in 5% CO_2_ with 95% air at 37°C.

### 2.2 Quantitative RT‐PCR assay

For fresh frozen tumor tissues or cells, total RNA was extracted using TRIzol^™^ reagent (Life Technologies, 15596026). For FFPE samples, a RecoverAll^™^ Total Nucleic Acid Isolation Kit (Life Technologies, AM1975) was used to extract total RNA according to the manufacturer's protocol. 1 μg total RNA per sample was reverse transcribed into cDNA using a PrimeScript^™^ RT Reagent Kit with gDNA Eraser (Takara, RR047A) according to the manufacturer's instructions. qRT‐PCR analysis was performed using the SYBR^®^ Premix Ex Taq^™^ Kit (Takara, RR420A) and fast real‐time fluorescent quantitative PCR system (Life Technologies; ABI 7900HT).

For fresh frozen tumor tissues or cells samples, primers for qRT‐PCR were as follows: DSCAM‐AS1‐F, 5′‐GTGACACAGCAAGACTCCCT‐3′ and DSCAM‐AS1‐R, 5′‐GATCCGTCGTCCATCTCTGT‐3′; and GAPDH‐F, 5′‐ TGACCCCTTCATTGACCTCA ‐3′ and GAPDH‐R, 5′‐ GGACTCCACGACGTACTCAG ‐3′.

To avoid bias caused by RNA degradation in FFPE samples,^11^ we designed relatively short amplicons (product length 60‐90 bp) as follows: DSCAM‐AS1‐SF, 5′‐ AGAGCGAAACCCCATCTCAA ‐3′ and DSCAM‐AS1‐SR, 5′‐ GATCCGTCGTCCATCTCTGT ‐3′; B2M‐SF, 5′‐ ACTCTCTCTTTCTGGCCTGG‐3′ and B2M‐SR, 5′‐ TCTCTGCTGGATGACGTGAG ‐3′. GAPDH‐SF, 5′‐ GAGTCCACTGGCGTCTTCA‐3′ and GAPDH‐SR, 5′‐ GGCAGAGATGATGACCCTTT ‐3′; RPLP0‐SF, 5′‐ TGGAAAACAACCCAGCTCTG ‐3′ and RPLP0‐SR, 5′‐ GGTGAACACAAAGCCCACAT ‐3′. To reduce the bias caused by one reference gene, 3 housekeeping genes (B2M, GAPDH, and RPLP0) were used as reference genes. The average Δ*C*_*T*_ value of the 3 housekeeping genes was used to normalize the expression level of DSCAM‐AS1. The annealing temperature of all the qPCR experiments was set as 60°C. All experiments were performed and analyzed in triplicate.

### 2.3 Transient transfection

Chemically synthesized siRNAs were purchased from RiboBio (Guangzhou, China). siRNA target sequences for DSCAM‐AS1 were as follows: si‐DSCAM‐AS1‐1, 5′‐GGAGATCACAGCCAAGGAA‐3′ and si‐DSCAM‐AS1‐2, 5′‐GCATGACATACTCATCCAT‐3′. For RNA transfection, 3 × 10^5^ (MCF‐7) or 5 × 10^5^ (T‐47D) cells were seeded onto 6‐well plates. Twenty‐four hours after seeding, 60 nmol/L siRNA was transfected using Lipofectamine^®^ RNAiMAX (ThermoFisher Scientific, 13778150) and Gibco^™^ Opti‐MEM (ThermoFisher Scientific, 51985042) according to the manufacturer's recommendations.

### 2.4 Cell proliferation and colony formation assays

Twenty‐four hours after transfection, cells were seeded onto 96‐well plates at a density of 3000 (MCF‐7) or 5000 (T‐47D) cells per well. Cells were examined using a CCK8 kit (Dojindo; CK04) every 2 days. Three wells were measured per condition.

For the colony formation assay, 3000 (MCF‐7) or 5000 (T‐47D) cells per well were seeded onto 6‐well plates. Two weeks later, cells were fixed with methyl alcohol, stained with crystal violet, and scored for colony formation manually from 3 replicate wells. Cell cluster larger than 120 μm in diameter (16 cells) was considered as a colony.

### 2.5 Flow cytometry

Forty‐eight hours after siRNA transfection, MCF‐7 and T‐47D cells were harvested and divided into 2 parts. Cellular apoptosis was tested using the Alexa Fluor 488 Annexin V/Dead Cell Apoptosis Kit (Life Technologies; V13241 and V13245). DNA was purified using RNase A (Thermo Fisher; 12091021) with a concentration of 1:1000 and cell cycle arrest was evaluated using propidium iodide (Invitrogen; P3566; 1 mg/mL) followed by flow cytometry (FC500; Beckman Coulter) as previously described.^12^ Fluorescence‐activated cell sorting (FACS) gating strategy was showed in Figure S3. Three biological replicates were performed before data analysis.

### 2.6 RNA‐seq analysis

MCF‐7 cells were plated onto 6‐well plates and transfected with si‐NC, si‐DSCAM‐AS1‐1, or si‐DSCAM‐AS1‐2 the following day. Forty‐eight hours after transfection, total RNA was extracted. RNA integrity was validated using denaturing gel electrophoresis and ethidium bromide (EtBr) staining. The total RNA samples (1 μg) were treated with VAHTS mRNA Capture Beads (Vazyme, Nanjing, China; N401‐01) to enrich polyA+ RNA. RNA‐seq libraries were prepared using a VAHTS mRNA‐seq v2 Library Prep Kit for Illumina (Vazyme, Nanjing, China; NR601‐01) according to the manufacturer's instructions. The libraries were sequenced by an Illumina sequencing platform on a 150 bp paired‐end run. Sequencing reads were aligned using the spliced read aligner HISAT2. The Ensembl human genome assembly (Genome Reference Consortium GRCh38) was used as the reference genome.

### 2.7 Patients and samples

Female patients pathologically diagnosed with infiltrating ductal breast carcinoma after tumor resection from January 2007 to December 2011 at Fudan University Shanghai Cancer Center (FUSCC) were recorded. ER positivity, which was validated by immunohistochemistry (IHC) in the pathology department at our hospital, was defined as luminal breast cancer. A total of 399 luminal breast cancer patients were included in the study. Patients’ clinicopathologic characteristics were collected, and all patients were followed up until March 2017. Disease‐free survival (DFS) was calculated from the time of surgery to the last follow‐up or event (relapse or death). Patient tumor samples were fixed with formalin, and embedded in paraffin immediately after resection and properly preserved. Sample slices containing more than 75% tumor cells were used for total RNA extraction.

### 2.8 Statistical analysis

Two‐tailed Student's *t* test was used to analyze the significance of difference among experimental groups. Log‐rank tests were applied to determine univariate prognostic factors. Kaplan‐Meier plots were used to draw survival curves. Factors with *P < *.2 in the univariate analysis were further analyzed using a multivariate Cox proportional hazards model to estimate the independent effects on DFS. Two‐tailed *P*‐values <.05 were considered statistically significant. All statistical analyses were conducted using SPSS 22.0 (SPSS, Chicago, IL, USA).

## 3 RESULTS

### 3.1 DSCAM‐AS1 regulates cell proliferation and colony formation by inducing the G_1_/S transition

**Figure 1:**
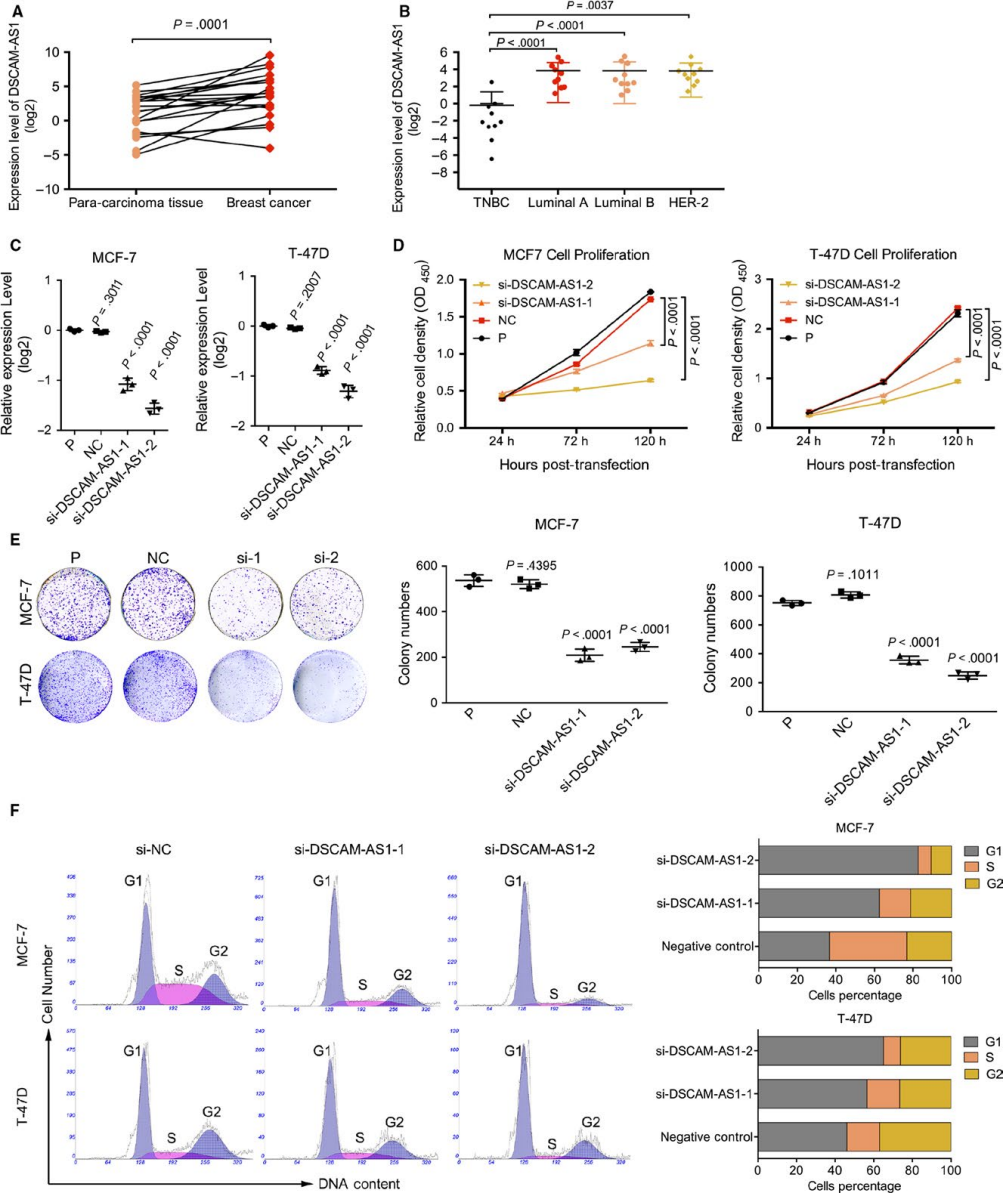
DSCAM‐AS1 regulates cell proliferation and colony formation by inducing the G1/S transition. A, DSCAM‐AS1 was highly expressed in 21 paired breast tumors and para‐carcinoma fresh frozen tissues by qRT‐PCR. Error bars represent the SD of 3 experimental replicates. B, DSCAM‐AS1 was highly expressed in luminal and Her‐2 overexpression subtypes of breast cancer among 40 fresh frozen breast cancer tissues by qRT‐PCR. Error bars represent the SD of 3 experimental replicates. C, Interference efficiency of siRNAs by qRT‐PCR targeting DSCAM‐AS1 in MCF‐7 and T‐47D cell lines. Error bars represent the SD of 3 biological replicates. D, CCK8 proliferation assay following knockdown of DSCAM‐AS1 by siRNAs showed reduced cell proliferation in MCF‐7 and T‐47D cell lines. Error bars represent the SD of 3 biological replicates. E, Knockdown of DSCAM‐AS1 by siRNAs reduced the cell clonality in MCF‐7 and T‐47D cell lines. Error bars represent the SD of 3 biological replicates. F, Knockdown of DSCAM‐AS1 by siRNAs induced G_1_/S cell cycle arrest in MCF‐7 and T‐47D cell lines. Error bars represent the SD of 3 biological replicates

In previous studies, DSCAM‐AS1 was screened and validated using public RNA‐seq databases, such as The Cancer Genome Atlas (TCGA) and MiTranscriptome.^13^ Thus, we first validated the expression distribution of DSCAM‐AS1 in fresh frozen samples. Twenty‐one pairs of fresh frozen luminal breast cancer samples and adjacent normal tissues were examined to detect DSCAM‐AS1 expression. DSCAM‐AS1 was highly expressed in breast cancer (Figure 1A). We also determined the distribution of DSCAM‐AS1 in different breast cancer subtypes. DSCAM‐AS1 was highly expressed in luminal and Her‐2 overexpression breast cancers but not in TNBC (Figure 1B), indicating that it plays important roles in ER+ breast tumors.

Next, we knocked down DSCAM‐AS1 using 2 siRNAs in 2 luminal breast cancer cell lines, MCF‐7 and T‐47D (Figure 1C). Down‐regulation of DSCAM‐AS1 significantly inhibited cell proliferation and colony formation in both cell lines (Figure 1D,E). Flow cytometric analysis revealed the growth inhibition caused by G_1_/S arrest (Figure 1F), not apoptosis (Figure S1). Hence, DSCAM‐AS1 regulates cell proliferation and colony formation by inducing the G1/S transition.

### 3.2 DSCAM‐AS1 participates in crucial biological processes

**Figure 2:**
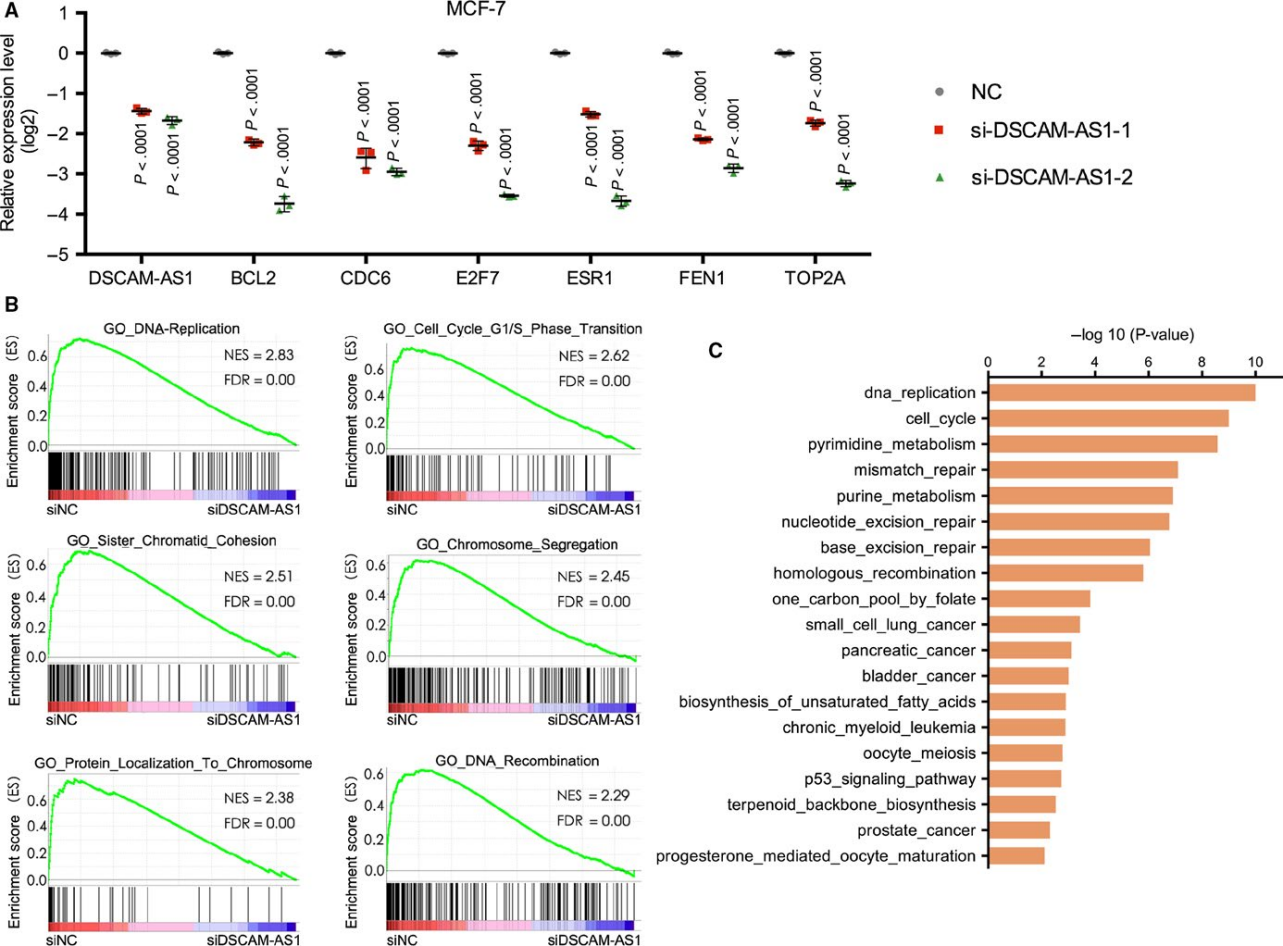
RNA‐seq analysis revealed that DSCAM‐AS1 is related to important cancer‐related biological processes. A, qRT‐PCR was used to validate the expression levels of genes related to tumor proliferation. Error bars represent the SD of 3 biological replicates. B, Gene set enrichment analysis (GSEA) of Gene Ontology (GO) revealed that the most significantly enriched gene sets were related to DNA replication, the G1/S phase transition, sister chromatid cohesion, chromosome segregation, protein localization to the chromosome and DNA recombination. C, Kyoto Encyclopedia of Genes and Genomes (KEGG) pathway analysis revealed that DSCAM‐AS1 is highly correlated with DNA replication, cell cycle, pyrimidine metabolism, mismatch repair, and other cancer‐related pathways

To further clarify the mechanism of DSCAM‐AS1, we performed RNA‐seq analysis with a control and 2 DSCAM‐AS1 knockdown MCF‐7 cell lines using siRNA. A total of 1283 genes were down‐regulated, and 922 genes were up‐regulated (*P* < .05; fold change ≥1.5). Several cell cycle‐related genes were examined to validate the reliability of the RNA‐seq results by qRT‐PCR. As was shown in Figure 2A, BCL2, CDC6, E2F7, ESR1, FEN1, and TOP2A were significantly down‐regulated in DSCAM‐AS1 knockdown cells. Gene set enrichment analysis (GSEA) of Gene Ontology (GO) results revealed that the most significantly enriched gene sets were related to DNA replication, the G1/S phase transition, sister chromatid cohesion, chromosome segregation, protein localization to the chromosome and DNA recombination (Figure 2B). Kyoto Encyclopedia of Genes and Genomes (KEGG) pathway analysis revealed that DSCAM‐AS1 was highly correlated with DNA replication, cell cycle, pyrimidine metabolism, mismatch repair and other cancer‐related pathways (Figure 2C). We also analyzed the potentially related miRNAs of DSCAM‐AS1. As shown in Figure S2, gene sets related to miR‐382, miR‐183, and miR‐99 were enriched in DSCAM‐AS1‐knockdown MCF‐7 cells. miR‐382^14^ and miR‐183^15^, ^16^, ^17^ affect tumor metastasis and invasion, while miR‐99 regulates the DNA damage response.^18^ Further experiments are needed to validate the relationships between DSCAM‐AS1 and these miRNAs. Together, these results highlight the crucial role of DSCAM‐AS1 in cell cycle and cancer progression.

### 3.3 DSCAM‐AS1 is an independent prognostic factor in luminal breast cancer patients treated with endocrine therapy

Although the effects of DSCAM‐AS1 in breast cancer cell proliferation, epithelial‐mesenchymal transition, and tamoxifen resistance have been researched, whether DSCAM‐AS1 affects patient prognosis remains unclear.^9^, ^10^ Yashar analyzed the survival rate of a TCGA breast cancer cohort on ER‐positive samples, but no significant difference in survival was found between patients with high and low expression of DSCAM‐AS1.^10^ Considering the imperfect follow‐up of the TCGA cohort, we cannot exclude the probability of false negative results. Hence, a longer follow‐up clinical trial that incorporates more complete clinicopathologic data, such as endocrine therapy information, is necessary.

In the current study, a total of 399 luminal breast cancer patients were enrolled, with a median follow‐up time of 58.91 months (interquartile range, 42.90‐70.65) and 72 events recorded (Table 1). The median age was 48 years (interquartile range, 42‐58 years). All patients underwent tumor resection. In total, 165 (41.35%) patients received radiotherapy, and 342 (85.71%) patients were treated with chemotherapy. With a cut‐off value of 14.18 (Δ*C*_*T*_ value), DSCAM‐AS1 was highly expressed in 239 patients and weakly expressed in 160 patients. No clinicopathologic characteristic was correlated with DSCAM‐AS1 expression in either the total cohort or the 2 subgroups.

**Table 1:** Clinicopathologic characteristics of 3 sets of luminal breast cancer patients according to DSCAM‐AS1 expression

Clinicopathologic characteristics	Total patients				Endocrine therapy group				No endocrine therapy group			
	DSCAM‐AS1 expression		χ^2^	*P*	DSCAM‐AS1 expression		χ^2^	*P*	DSCAM‐AS1 expression		χ^2^	*P*
	Low n (%)	High n (%)			Low n (%)	High n (%)			Low n (%)	High n (%)		
Age												
≤50	92 (41.82)	128 (58.18)	0.603	.438	74 (41.11)	106 (58.89)	0.020	.888	18 (45.00)	22 (55.00)	1.60	.206
>50	68 (37.99)	111 (62.01)			52 (40.31)	77 (59.69)			16 (32.00)	34 (68.00)		
Size												
≤2 cm	77 (40.74)	112 (59.26)	0.061	.804	63 (42.00)	87 (58.00)	0.181	.671	14 (35.90)	25 (64.10)	0.104	.748
>2 cm	83 (39.52)	127 (60.48)			63 (39.62)	96 (60.38)			20 (39.22)	31 (60.78)		
Grade												
I	8 (50.00)	8 (50.00)	1.151	.562	7 (53.85)	6 (46.15)	2.408	.300	1 (33.33)	2 (66.67)	0.684	.710
II	100 (40.98)	144 (59.02)			84 (42.64)	113 (57.36)			16 (34.04)	31 (65.96)		
III	52 (37.41)	87 (62.59)			35 (35.35)	64 (64.65)			17 (42.50)	23 (57.50)		
Positive lymph nodes												
0	84 (39.25)	130 (60.75)	1.434	.488	65 (40.12)	97 (59.88)	0.744	.689	19 (36.54)	33 (63.46)	0.833	.659
1‐3	42 (45.16)	51 (54.84)			34 (44.74)	42 (55.26)			8 (47.06)	9 (52.94)		
>3	34 (36.96)	58 (63.04)			27 (38.03)	44 (61.97)			7 (33.33)	14 (66.67)		
ER												
+	14 (28.00)	36 (72.00)	5.061	.080	10 (28.57)	25 (71.43)	4.298	.117	4 (26.67)	11 (73.33)	0.993	.609
++	33 (48.53)	35 (51.47)			25 (51.02)	24 (48.98)			8 (42.11)	11 (57,89)		
+++	113 (40.21)	168 (59.79)			91 (40.44)	134(59.56)			22 (39.29)	34 (60.71)		
PR												
−	21 (46.67)	24 (53.33)	1.714	.634	11 (44.00)	14 (56.00)	2.011	.570	10 (50.00)	10 (50.00)	2.855	.415
+	31 (36.47)	54 (63.53)			23 (34.33)	44 (65.67)			8 (44.44)	10 (55.56)		
++	48 (42.48)	65 (57.52)			39 (45.35)	47 (54.65)			9 (33.33)	18 (66.67)		
+++	60 (38.46)	96 (61.54)			53 (40.46)	78 (59.54)			7 (28.00)	18 (72.00)		
Ki‐67												
≤20	77 (40.96)	111 (59.04)	0.137	.711	57 (40.43)	84 (59.57)	0.006	.940	20 (42.55)	27 (57.45)	0.954	.329
>20	81 (39.13)	126 (60.87)			67 (40.85)	97 (59.15)			14 (32.56)	29 (67.44)		
Radiotherapy												
Yes	62 (37.58)	103 (62.42)	0.746	.388	54 (38.30)	87 (61.70)	0.660	.417	8 (33.33)	16 (66.67)	0.275	.600
No	98 (41.88)	136 (58.12)			72 (42.86)	96 (57.14)			26 (39.39)	40 (60.61)		
Chemotherapy												
Yes	139 (40.64)	203 (59.36)	0.294	.588	117 (51.32)	171 (48.68)	0.040	.841	22 (40.74)	32 (59.26)	0.504	.478
No	21 (36.84)	36 (63.16)			9 (42.86)	12 (57.14)			12 (33.33)	24 (66.67)		
Endocrine therapy												
Yes	126 (40.78)	183 (59.22)	0.261	.609								
No	34 (37.78)	56 (62.22)										

**Figure 3:**
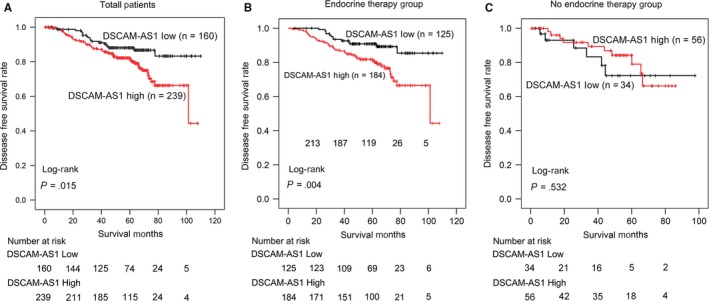
Kaplan‐Meier plots revealed the effect of DSCAM‐AS1 expression in luminal breast cancer disease‐free survival (DFS). A, High DSCAM‐AS1 expression was related to a worse DFS in 399 luminal breast cancer patients (*P* = .015). B, High DSCAM‐AS1 expression correlated with a worse DFS in 309 luminal breast cancer patients treated with endocrine therapy (*P* = .004). C, DSCAM‐AS1 expression had no effect on DFS in 90 luminal breast cancer patients without endocrine therapy (*P* = .532)

Next, we investigated the relationships between DSCAM‐AS1 expression and other clinicopathologic characteristics with disease‐free survival (DFS). In the total cohort, univariate survival analysis revealed that characteristics, including tumor size (*P* = .005), grade (*P* = .004), positive lymph node number (*P* < .0001), and ki‐67 (*P* = .012), correlated with prognosis (Table 2). High expression of DSCAM‐AS1 correlated with a shorter DFS (*P* = .015, Figure 3A). Prognostic factors with *P* values less than .2 were included in the multivariate analysis. Positive lymph node number (hazard ratio [HR], 2.19; 95% confidence interval [CI], 1.64‐2.93; *P* < .0001) and ki‐67 (HR, 1.81; 95% CI, 1.10‐3.00; *P* = .020) were identified as independent factors affecting DFS. DSCAM‐AS1 was also identified as an independent factor in the total cohort (HR, 1.79; 95% CI, 1.04‐3.06; *P* = .035).

**Table 2:** Univariate and multivariate disease free survival analysis of the 3 sets of luminal breast cancer patients

Clinicopathologic characteristics	Total patients			Endocrine therapy group			No endocrine therapy group		
	Univariate analysis	Multivariate analysis		Univariate analysis	Multivariate analysis		Univariate analysis	Multivariate analysis	
	*P*	HR (95% CI)	*P*	*P*	HR (95% CI)	*P*	*P*	HR (95% CI)	*P*
Age	.362			.642			.291		
Size	**.005**	1.46 (0.88‐2.42)	.145	**.017**	1.46 (0.83‐2.59)	.191	.148	1.34 (0.45‐3.96)	.452
Grade	**.004**	1.50 (0.95‐2.36)	.084	**.001**	1.87 (1.10‐3.18)	**.022**	.422		
Positive lymph nodes	**<.0001**	2.19 (1.64‐2.93)	**<.0001**	**<.0001**	2.05 (1.47‐2.85)	**<.0001**	**.002**	2.51 (1.39‐4.54)	**.002**
ER	.266			.631			.428		
PR	.123	0.81 (0.63‐1.05)	.112	.226			.528		
Ki‐67	**.012**	1.81 (1.10‐3.00)	**.020**	.051	1.58 (0.90‐2.80)	.112	.088	2.90 (0.99‐8.50)	.053
Radiotherapy	.345			.203			.979		
Chemotherapy	.258			.883			.262		
Endocrine therapy	.152	0.65 (0.36‐1.16)	.146						
DSCAM‐AS1	**.015**	1.79 (1.04‐3.06)	**.035**	**.004**	2.27 (1.19‐4.34)	**.013**	.532		

Boldface represent *P* value less than .05.

As DSCAM‐AS1 has been reported to induce tamoxifen resistance, we next investigated whether DSCAM‐AS1 affects the prognosis of patients treated with endocrine therapy. Among the 399 patients examined, 309 (77.44%) received endocrine therapy and were included in the endocrine therapy group. The other 90 (22.56%) patients were included in the no endocrine therapy group. In the endocrine therapy group, DSCAM‐AS1 was weakly expressed in 125 (40.45%) patients and highly expressed in 184 (59.55%) patients. DSCAM‐AS1 (*P* = .004), tumor size (*P* = .017), grade (*P* = .001), and positive lymph node number (*P* < .0001) were determined to affect DFS in the univariate analysis. Patients with high DSCAM‐AS1 expression had a shorter DFS (Figure 3B). In the multivariate analysis, DSCAM‐AS1 (HR, 2.27; 95% CI, 1.19‐4.34; *P* = .013), grade (HR, 1.87; 95% CI, 1.10‐3.18; *P* = .022) and positive lymph node number (HR, 2.05; 95% CI, 1.47‐2.85; *P* < .0001) were identified as independent prognostic factors.

However, in the no endocrine therapy group, DSCAM‐AS1 expression showed no effect on patients DFS (*P* = .532). Considering the small sample size (n = 90), our conclusion should be confirmed in a larger cohort with a longer follow‐up.

## 4 DISCUSSION

In this study, we revealed that DSCAM‐AS1 regulates cell proliferation and colony formation by inducing the G1/S transition. RNA‐seq analysis demonstrated that DSCAM‐AS1 participates in crucial biological processes, including DNA replication, the G1/S phase transition, sister chromatid cohesion, chromosome segregation, protein localization to the chromosome, and DNA recombination. DSCAM‐AS1 is a prognostic factor in patients with luminal breast cancer. In luminal breast cancer, patients treated with endocrine therapy, high expression of DSCAM‐AS1 may represent a poor independent factor that affects DFS.

lncRNA are defined as transcripts longer than 200 nucleotides that are not translated into protein.^19^ Although DSCAM‐AS1 is a newly reported cancer‐related lncRNA, it was first described as a mRNA in 2002, when it was determined to be expressed at a higher level in human breast cancer specimens than in normal human breast and benign lesions.^8^ It then lost attention until in 2014, when Weihong et al reported that DSCAM‐AS1 was differentially expressed in lung cancer. Over the next 2 years, DSCAM‐AS1 was found to play important roles in luminal breast cancer.^9^, ^10^ While DSCAM‐AS1 is highly expressed in ER+ and HER‐2 overexpressed breast cancer cell lines, it is mainly enriched in luminal breast cancer samples. Remarkably, DSCAM‐AS1 classifies as the most abundant lncRNA in luminal breast cancer cell lines.^9^ Additionally, it is directly regulated by ERα, which makes it different from other lncRNAs in luminal breast cancer.

Previous studies revealed that DSCAM‐AS1 affects tumor proliferation and invasion, although the exact mechanism remains unclear. The only reported protein that directly interacts with DSCAM‐AS1 is hnRNPL, which mainly exists in the cytoplasm to promote tumor invasion. In the current study, flow cytometry confirmed that DSCAM‐AS1 induces tumor growth by promoting the G_1_/S transition. RNA‐seq analysis revealed that DNA replication and cell division are correlated with DSCAM‐AS1 expression, indicating that promoting proliferation is its main function and that the role of DSCAM‐AS1 in the nucleus may be more important. Considering the vital pathways associated with DSCAM‐AS1 in the nucleus, it may be necessary to identify the proteins that induce proliferation. Although the flow cytometry experiments did not show any effect of DSCAM‐AS1 on apoptosis, knocking down DSCAM‐AS1 does influence apoptosis related genes such as BCL2. Accounting for the multiple roles of DSCAM‐AS1 which the GO and GSEA analysis has indicated, the fact that DSCAM‐AS1 has an indirect or little influence on apoptosis may be reasonable.

Additionally, it is interesting that ESR1 was downregulated when DSCAM‐AS1 was knockdown. As was reported, DSCAM‐AS1 was regulated by ERα. If ERα is also regulated by DSCAM‐AS1, they may form a positive feedback, which means ERα and DSCAM‐AS1 can maintain their high expression each other in luminal (ER+) breast cancer. This may also explain why DSCAM‐AS1 is weakly expressed in TNBC.

Finally and most importantly, for the first time, we defined the clinical significance of DSCAM‐AS1. In luminal breast cancer patients, high DSCAM‐AS1 expression is an independent factor of poor DFS. In patients treated with endocrine therapy, the significance becomes more obvious. Hence, endocrine therapy resistance may be a momentous function for DSCAM‐AS1.

## 5 CONCLUSIONS

In conclusion, luminal breast cancer patients with high DSCAM‐AS1 expression level are inclined to be resistant to endocrine therapy. On the other hand, DSCAM‐AS1 is a promising clinical therapeutic target that may prolong survival in luminal breast cancer patients treated with endocrine therapy.

## ETHICS APPROVAL AND CONSENT TO PARTICIPATE

This study was approved by the Fudan University Shanghai Cancer Center (FUSCC) Ethics Committee, and each patient provided informed written consent.

## CONSENT TO PUBLISH

Not applicable.

## AVAILABILITY OF DATA AND MATERIALS

All raw data of RNA‐seq generated and/or analyzed in this study are available from the corresponding author on reasonable request. The sequences for primers and si‐RNA target sequences are available in the Methods section.

## CONFLICT OF INTERESTS

The authors declare that they have no competing interests.
