# Supplementary figures and images for: DSCAM‐AS1 regulates the G1/S cell cycle transition and is an independent prognostic factor of poor survival in luminal breast cancer patients treated with endocrine therapy
Source: Cancer Med. 2018 Nov 14;7(12):6137–46. doi: 10.1002/cam4.1603 (PMC6308059; doi:10.1002/cam4.1603)

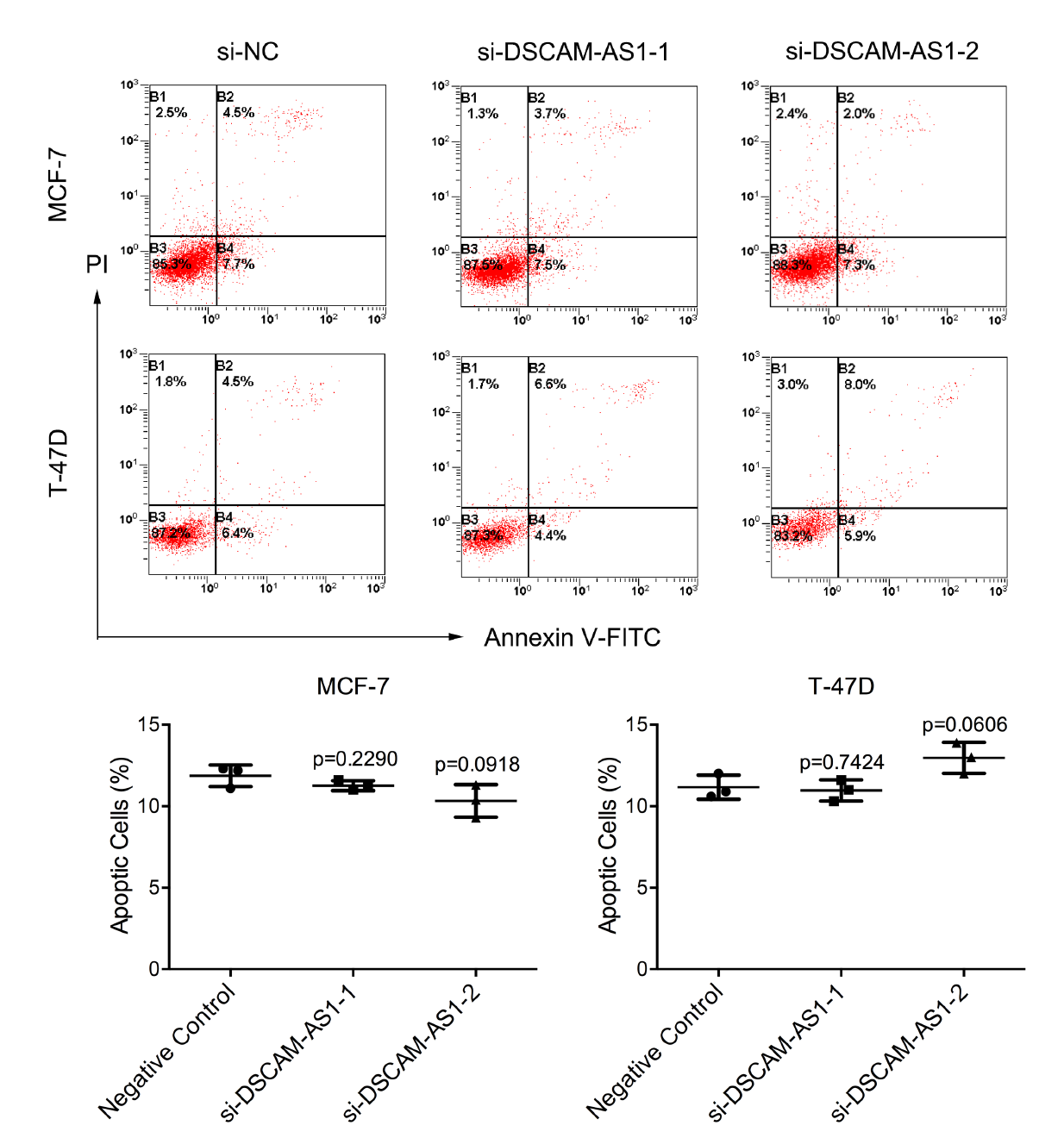

Supplement: Supplementary file 1 [file CAM4-7-6137-s001.tif]

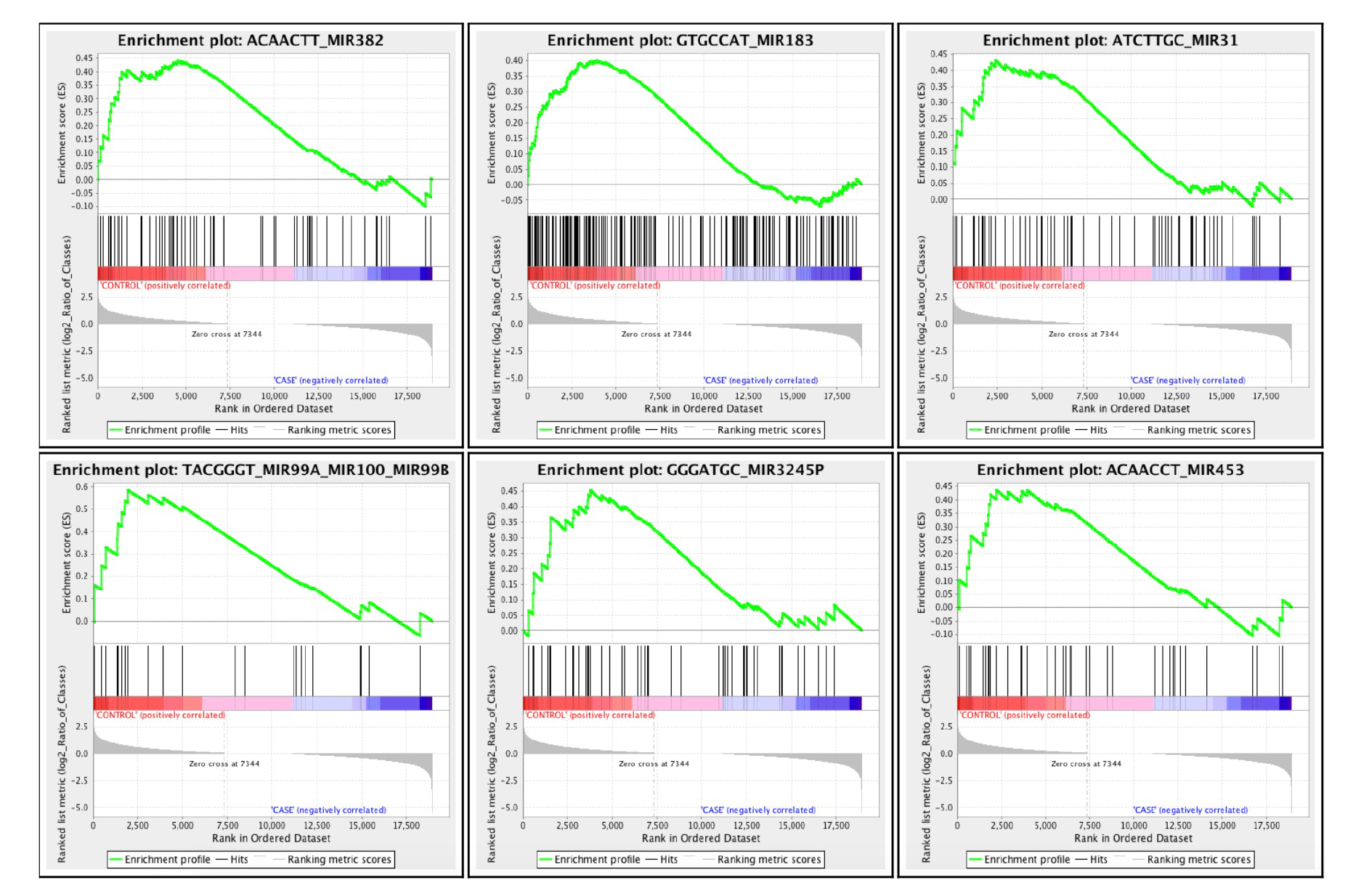

Supplement: Supplementary file 2 [file CAM4-7-6137-s002.tif]

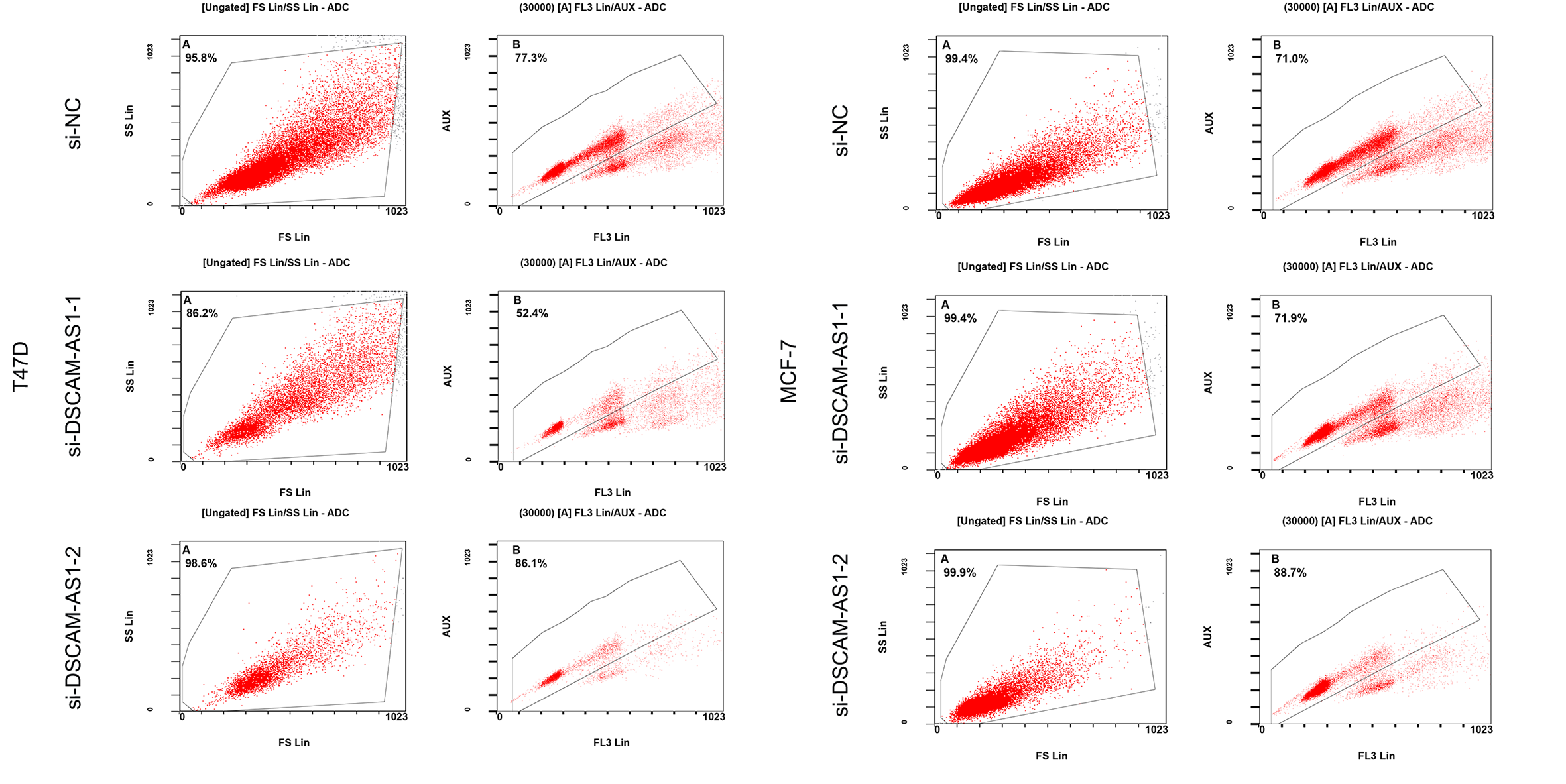

Supplement: Supplementary file 3 [file CAM4-7-6137-s003.tif]
