# Supplementary material for: DSCAM‐AS1 regulates the G1/S cell cycle transition and is an independent prognostic factor of poor survival in luminal breast cancer patients treated with endocrine therapy
Source: Cancer Med. 2018 Nov 14;7(12):6137–46. doi: 10.1002/cam4.1603 (PMC6308059; doi:10.1002/cam4.1603)
Supplement: Supplementary file 4 [file CAM4-7-6137-s004.docx]

Fig. S1 Knockdown of DSCAM-AS1 by siRNAs had no effect on apoptosis in MCF-7 or T-47D cell lines. Error bars represent the SD of three biological replicates.

Fig. S2 Gene set enrichment analysis (GSEA) revealed that gene sets related to miR-382, miR-183, and miR-99 were enriched in DSCAM-AS1-knockdown MCF-7 cells.

Fig. S3 Fluorescence activated cell sorting (FACS) gating strategy for figure 1F.
